# Dihydrotanshinone I enhanced BRAF mutant melanoma treatment efficacy by inhibiting the STAT3/SOX2 signaling pathway

**DOI:** 10.3389/fonc.2025.1429018

**Published:** 2025-01-29

**Authors:** Xing Luo, Yi Duan, Jinwei He, CongGai Huang, Jun Liu, Yifan Liu, Mengdei Xu, Qiong Dai, Zhihui Yang

**Affiliations:** ^1^ Department of Pathology, the Affiliated Hospital of Southwest Medical University, Luzhou, Sichuan, China; ^2^ School of Basic Medical Sciences, Southwest Medical University, Luzhou, Sichuan, China; ^3^ Clinical School of Medicine, Southwest Medical University, Luzhou, Sichuan, China; ^4^ Department of Human Anatomy, School of Basic Medical Sciences, Southwest Medical University, Luzhou, Sichuan, China

**Keywords:** BRAF mutation, dihydrotanshinone I, melanoma, MAPK pathway, STAT3

## Abstract

**Background:**

The Food and Drug Administration has approved the Serine/threonine-protein kinase B-raf (BRAF) inhibitor and Mitogen-activated extracellular signal-regulated kinase (MEK) inhibitor combo as the first-line treatment for individuals with metastatic melanoma, although the majority of these patients exhibit primary or secondary drug resistance in the clinic. Dihydrotanshinone I (DHT) is a lipophilic compound extracted from the root of Salvia miltiorrhiza that has been linked to multiple antitumor activities. In this study, we investigated the effect of dihydrotanshinone I on the MAPK pathway inhibitor resistance of BRAF mutant malignant melanoma.

**Method:**

After treating A375, A375R, and A2058 cells with DHT or a combination of DHT and BRAF/MEK inhibitors, WB and Real-Time RT-qPCR were used to confirm the activation of the MAPK and STAT3/SOX2 pathways. CCK-8 was used to assess cell viability, while flow cytometry was used to identify apoptosis. In addition, mice were inoculated with A375 cells to establish a model of tumour formation, and various drug groups and treatment models were utilized. The diameter and weight of tumours in each group were then measured, and IHC and HE staining were used to assess the expression of two pathways and cytotoxicity, respectively.

**Results:**

This study found that DHT directly interacts with STAT3 protein and it can stop the feedback activation of the STAT3/SOX2 pathway caused by the use of MAPK pathway inhibitors. In addition, the combination of DHT and BRAF/MEK inhibitors can inhibit the proliferation and growth of BRAF mutant melanoma cells and primary and secondary drug-resistant cells. Finally, we proved that the combined therapy of DHT and BRAF/MEK inhibitors is reliable and effective at animal and cell levels.

**Conclusion:**

In BRAF mutant melanoma cells, DHT suppresses the STAT3/SOX2 signaling pathway. Combining DHT, BRAF inhibitors, and MEK inhibitors can help treat treatment-resistant BRAF mutant melanoma cells. Experimental results both *in vitro* and *in vivo* have shown that the combination of DHT and an inhibitor of the MAPK pathway is safer and more successful than using an inhibitor of the MAPK pathway alone when treating BRAF mutant melanoma.

## 1 Introduction

Malignant melanoma, known as the most deadly kind of skin cancer, is caused by melanocytes. The incidence and fatality rate of melanoma are rising annually on a worldwide basis (1). The discovery of hotspot mutations in the BRAF gene, which codes for the serine/threonine protein kinase BRAF, has made it possible to identify inhibitors specific to these mutations and develop treatments aimed specifically at melanoma (2, 3). The FDA-approved RAF and MEK inhibitors may extend progression-free survival (PFS) in melanoma patients, and the combination of the two drugs is somewhat more advantageous than either drug alone (4). However, because acquired drug resistance emerges undoubtedly, melanoma patients do not achieve satisfactory results (5). Although immunotherapy has helped some melanoma patients, its usage is limited in advanced stages of the disease because of its high toxicity (6). It is crucial to look into the molecular causes of drug resistance in BRAF-mutated melanoma in order to develop more effective therapeutic strategies to improve the effectiveness of currently recommended treatment regimens and prevent the development of acquired drug resistance.

In our previous investigation, we found that inhibiting the MAPK pathway can reactivate signal transduction and transcription activator 3 (STAT3) in BRAF mutant melanoma cells (7). SRY-box2(SOX2) is an embryonic stem cell-expressed gene that keeps melanoma-initiating cells self-renewing and tumorigenic (8). In melanoma, STAT3 might be involved in regulating SOX2 (9). These findings imply that blocking the STAT3/SOX2 and MAPK pathways at the same time may be an effective treatment strategy for BRAF mutant melanoma. However, no research has demonstrated the security and viability of this combination therapy.

Dihydrotanshinone I (DHT), a fat-soluble substance derived from Salvia miltiorrhiza Bunge, is utilized extensively for its anti-inflammatory and cardiovascular protective properties (10, 11). DHT’s anti-tumor effectiveness has been the subject of numerous reports in recent years. For instance, it can successfully block the migration and epithelial-mesenchymal transition (EMT) of Patu8988 and PANC-1 cells, as well as cause death in these cells via the Hedgehog/Gli signal (12). DHT has reportedly been shown to cause MCF-7 and MDA-MB-231G1 to arrest and undergo apoptosis. And it has also been demonstrated that DHT can stop the growth of tumors in 4T1 allograft nude mice and prevent lung metastasis of breast cancer by preventing the formation of neutrophil extracellular traps (NETs) (13, 14). Additionally, DHT shows clear tumor growth suppression on oxaliplatin-resistant human HCT116 colorectal cancer cells *in vivo* and *in vitro (* 15, 16). Research has demonstrated that DHT can suppress STAT3 expression in esophageal squamous cell carcinoma and hepatocellular carcinoma (17, 18). However, the function and mechanism of DHT in melanoma remain unknown. Furthermore, it is unknown if DHT can prevent STAT3/SOX2 reactivation caused by MAPK pathway inhibitors in BRAF mutant melanoma. This study suggests using DHT and MAPK pathway inhibitors to treat refractory BRAF mutant melanoma. Furthermore, it was discovered that by concurrently blocking the MAPK/c-Myc/cyclin D1 and STAT3/SOX2 pathways, a combination of DHT and MAPK pathway inhibitors could accelerate the death of melanoma cells. Our study demonstrates the potential impact of DHT on BRAF mutant melanoma and offers a novel approach to treating drug resistance in melanoma.

## 2 Materials and methods

### 2.1 Cell lines and treatments

Human A375 and A2058 cells were donated by Dr. K. Zhao (Southwestern Medical University, Sichuan, China). Human proximaltubular epithelial cell line (HK-2) was gratefully presented by Prof. Bo Chen (Southwestern Medical University, Sichuan, China). Our previous research team administered a very high dose of Vemurafenib to A375 cells in order to rapidly screen the remaining cells. Following a 24-hour culture with 100 micron Vemurafenib, they were rinsed with PBS, cultivated for 21 days in fresh media devoid of Vemurafenib, then administered 5 micron Vemurafenib for 4 weeks. Lastly, their cell sensitivity was assessed to yield A375R cells. 10% fetal bovine serum (FBS) (BI, Israel) was added to DMEM (Gibco, Rockford, IL, USA) for the culture of A375, A375R, and A2058 cells. 10% FBS was added to 1640 (Gibco, Rockford, IL, USA) culture media, which was employed for HK-2.

### 2.2 Reagents and antibodies

The following drugs were bought: DHT from Manstead Biotechnology (Sichuan, China); vemurafenib, dabrafenib, cobimetinib, and trametinib from Selleckchem (Houston, TX, USA). A stock solution containing 10 μM of each inhibitor was created by dissolving it in DMSO. The following products were purchased: anti-STAT3, anti-phospho-STAT3, Anti-S0X2 (Abcam, Cambridge, UK); anti-CyclinD1, anti-c-Myc, anti-cleaved PARP (CST, MA, USA); anti-ERK1/2, anti-phospho-ERK1/2,anti-Tubulin, Anti-S0X2 (HUABIO, Zhejiang, China). The specific numbers of reagents and antibodies are listed in **Supplementary Table S** **1** and **S2**.

### 2.3 Western blot

Total protein was obtained from 6-cm dish-treated cells, and 15 μg of it was added to each well of a 10% SDS-PAGE gel for separation. Membranes made of polyvinylidene difluoride (PADF) (Millipore, Billerica, MA, USA) were electroblotted, sealed in 5% milk, and incubated with primary antibodies at different doses for a whole night at 4°C. After three TBST rinses, peroxidase-coupled secondary antibodies were allowed to incubate on the strips for one hour. After three TBST rinses, the bands were analyzed using the MiniChemi™ chemiluminescence imager from Sage Venture Technology Co (Beijing, China).

### 2.4 RNA extraction and RT-qPCR

Using Trizol reagent (Tiangen Biotech,Beijing,China), total RNA was extracted from the cells to measure the level of gene expression, and a reverse transcription kit (TaKaRa, Kyoto, Japan) was used to create cDNA. The expression of the SOX2, c-Myc, and Cyclin D1 genes was then measured in real time using SYBR dyes and primers. The degree of expression was contrasted with tubulin mRNA. Primers are listed in **Supplementary Table S3**.

### 2.5 Cell viability assay

Approximately 1000 cells per well were inoculated into each well of a 96-well microtitre plate. After 24 hours of drug-free culture, Various inhibitors were applied to A375, A375R, and A2058 cells for a duration of 72 hours. Then, in accordance with the directions, a 96-well plate was filled with cell counting kit 8 (bimake, TX, USA). Finally, an enzyme marker at 450 nm was used to assess absorbance.

### 2.6 Flow cytometric analysis

A375 cells were cultured overnight in 6CM dishes and then treated for 24 hours with various inhibitors. The FITC apoptosis detection kit (Annexin V, Dawjindao, Japan) was used to treat the cells in accordance with the instructions. Next, using flow cytometry (FACSCalibur, Becton-Dickinson), the percentage of apoptotic cells was determined.

### 2.7 Histological analysis and immunohistochemistry staining

The distinct tissues from the kidney, heart, and tumor were promptly preserved in phosphate solution containing 4% paraformaldehyde, after which they were dried, embedded in paraffin, and cut into slices. After being stored in a 60°C oven for 24 hours, tissue pieces were dewaxed with xylene and hydrated with an ethanol gradient (100%-70%). Hematoxylin-eosin (Baso, Zhuhai, China) was used to stain heart, liver, and kidney tissue sections. Following high-pressure thermal repair in a sodium citrate buffer with a concentration of 10 mmol/L (pH 6.0), it was allowed to cool to room temperature before being submerged in 3% H2O2 for ten minutes. After incubating the first antibody at room temperature for three hours, the second antibody needs to be incubated for thirty minutes. Lastly, dyeing should be done with 3,3’-diaminobenzidine (DAB).

### 2.8 *In vivo* studies

Male, four-week-old, naked Purchased from Chongqing Tengxinbier Experimental Animal Sales Co., Ltd. were BALB/c-mice devoid of the thymus gland. Every mouse was handled in accordance with the strategy that was authorized by Southwest Medical University’s Animal Care and Use Committee(SWMU20230911-002). The mice spent five days in conditions with regulated humidity and temperature prior to the experiment’s commencement. Each mouse had A375 (1 x 10^5^ cells) implanted subcutaneously into its right axilla. Mice were randomized into treatment groups until the tumor volume approached roughly 70 mm ± 10 mm^3^. Callipers were used every other day to measure the tumour volume. Each group’s average tumor volume is given in millimeters (mm^3^) using the formula (length× width 2)/2. After a two-week trial, mice were given an intraperitoneal injection of 1% pentobarbital sodium (50 mg/kg) based on their weight, which rendered them unconscious. The mice were then killed by breaking their necks. Immunohistochemical staining (IHC) and hematoxylin-eosin staining (H&E) were performed on tumor xenografts and internal organs. IHC was performed to assess p-STAT3, p-ERK, c-Myc, SOX2, and cyclin D1 expression, while HE was utilized to assess potential toxicity of the drugs.

### 2.9 Statistical analysis

The mean standard deviation (SD) was used to express all statistical date, which was subsequently examined with GraphPad Prism (GraphPad Software, California, USA). At least three separate runs of each experiment were completed. Students’ t-tests were used to gauge the results’ statistical significance.

## 3 Results

### 3.1 DHT specifically targets the SH2 domain of STAT3, and prevents BRAF inhibitor-induced compensatory activation of the STAT3/SOX2 pathway signals in BRAF mutant melanoma cells

**Figure 1:**
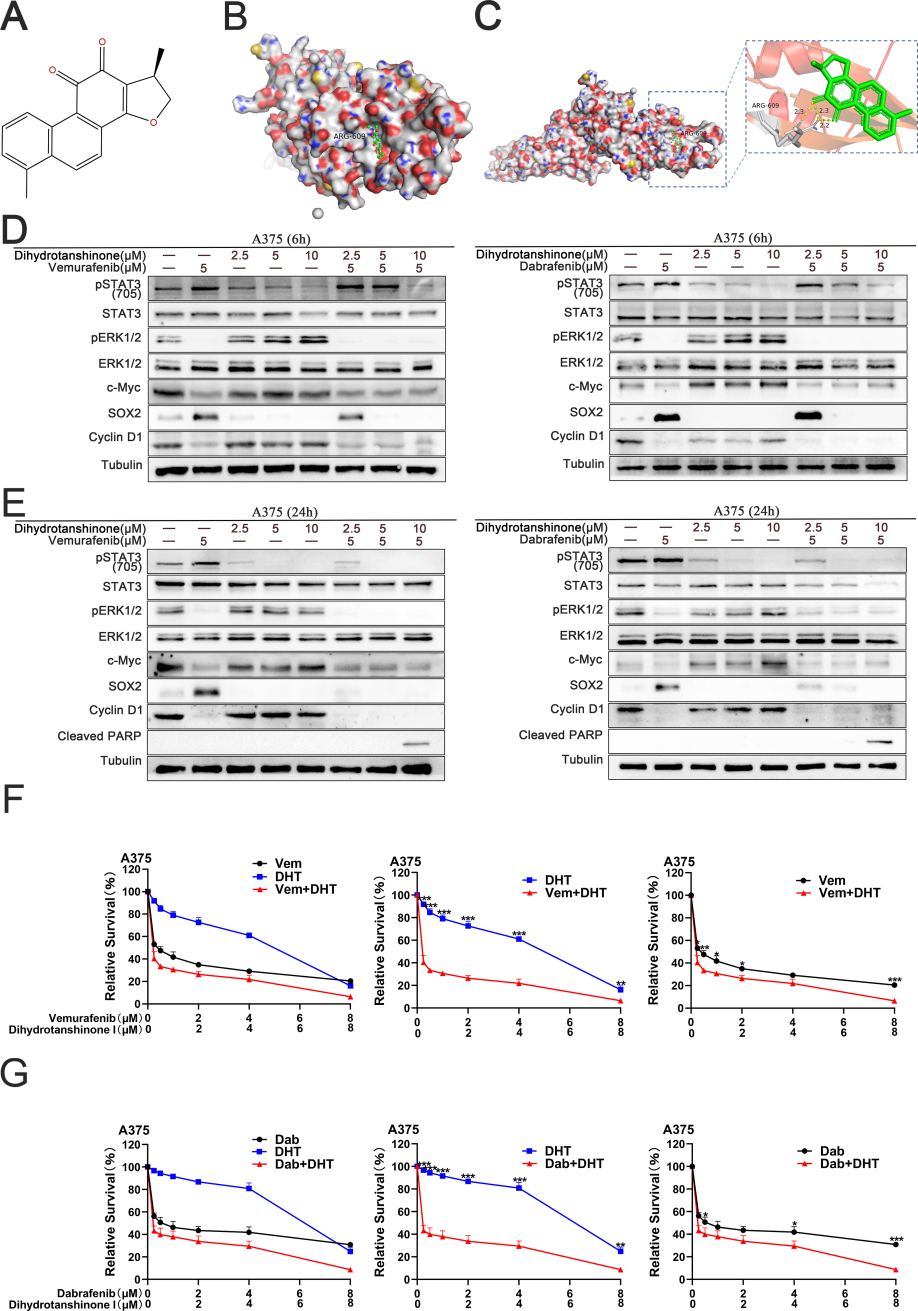
DHT directly binds to SH2 domain of STAT3 protein, blocking the compensatory activation of STAT3/SOX2 pathway signals caused by the application of BRAF inhibitors in BRAF mutant melanoma cells **(A)** The chemical structure of dihydrotanshinone I (PubChem ID: 11425923). **(B)** Surface representation of the intricate crystal structure of the DHT and STAT3 proteins’ SH2 domains (PDB ID: 6NJS). **(C)** By using computation docking, the expected interaction between the SH2 domain’s amino acid residues and DHT is displayed. Hydrogen bonds bind DHT and ARG609 together. N and O atoms are depicted on the surface of the STAT3 protein in blue and red, respectively. **(D)** Applying varying DHT concentrations to A375 cells that have been treated with BRAF inhibitors (vemurafenib or dabrafenib) for a period of 6 hours. Using tubulin as a loading control, western blotting analysis was used to measure the expression levels of the Cleaved PARP protein, MAPK/c-Myc/cyclin D1, and STAT3/SOX2 pathways. **(E)** Applying varying DHT concentrations to A375 cells that have been treated with BRAF inhibitors (vemurafenib or dabrafenib) for a period of 24 hours. **(F, G)** A375 cells were exposed to varying doses of either vemurafenib and DHT **(F)** or dabrafenib and DHT **(G)** for 72h. CCK8 tests were used to measure cell viability. Three duplicates of each experiment were run. Data are means ± SD. ***p < 0.001; **p < 0.01; *p < 0.05; Student’s t-test.

Initially, we utilized molecular docking simulation to predict and assess the interaction between STAT3 and DHT (**Figure 1A**). In the predicted binding conformation, DHT tightly bound the SH2 domain of STAT3, limiting STAT3 homodimer formation (**Figures 1B, C**). In additional experiments, the hypothesis that DHT prevents the compensatory stimulation of the STAT3 signal in cells of melanoma with a BRAF mutation and augments the lethal effect of a BRAF inhibitor alone was further evaluated. In A375 cells, treatment with a 5μM dose of a BRAF inhibitor (vemurafenib or dabrafenib) resulted in the inhibition of the p-ERK, c-Myc and Cyclin D1, and the reactivation of the STAT3 and SOX2 6 and 24 hours later. Similarly, when DHT was administered to A375 cells at dosages of 2.5, 5, and 10μM for 6 and 24 hours, respectively, the p-ERK, c-Myc and Cyclin D1 was triggered and the STAT3 and SOX2 was inhibited. Then, when DHT and BRAF inhibitors (vemurafenib or dabrafenib) are combined, the p-ERK, c-Myc, Cyclin D1,STAT3 and SOX2 can be inhibited concurrently. In addition, the conjunction of DHT and BRAF inhibitors increased the expression of cleaved PARP (**Figures 1D, E**). Lastly, we verify this hypothesis further by measuring the inhibition of survival using the quantitative CCK-8 method. Compared to A375 cells receiving a BRAF inhibitor (vemurafenib or dabrafenib) or DHT alone, all A375 cells handled with BRAF inhibitor (vemurafenib or dabrafenib) and DHT were significantly less vital (**Figures 1F, G**). In a nutshell, our results demonstrate that DHT targets STAT3 and suppresses the STAT3/SOX2 signaling pathway to reduce the activity of BRAF mutant melanoma cells.

### 3.2 BRAF mutant melanoma cells undergo more apoptosis when DHT is combined with BRAF and MEK inhibitors to block the MAPK and STAT3/SOX2 pathways simultaneously

**Figure 2:**
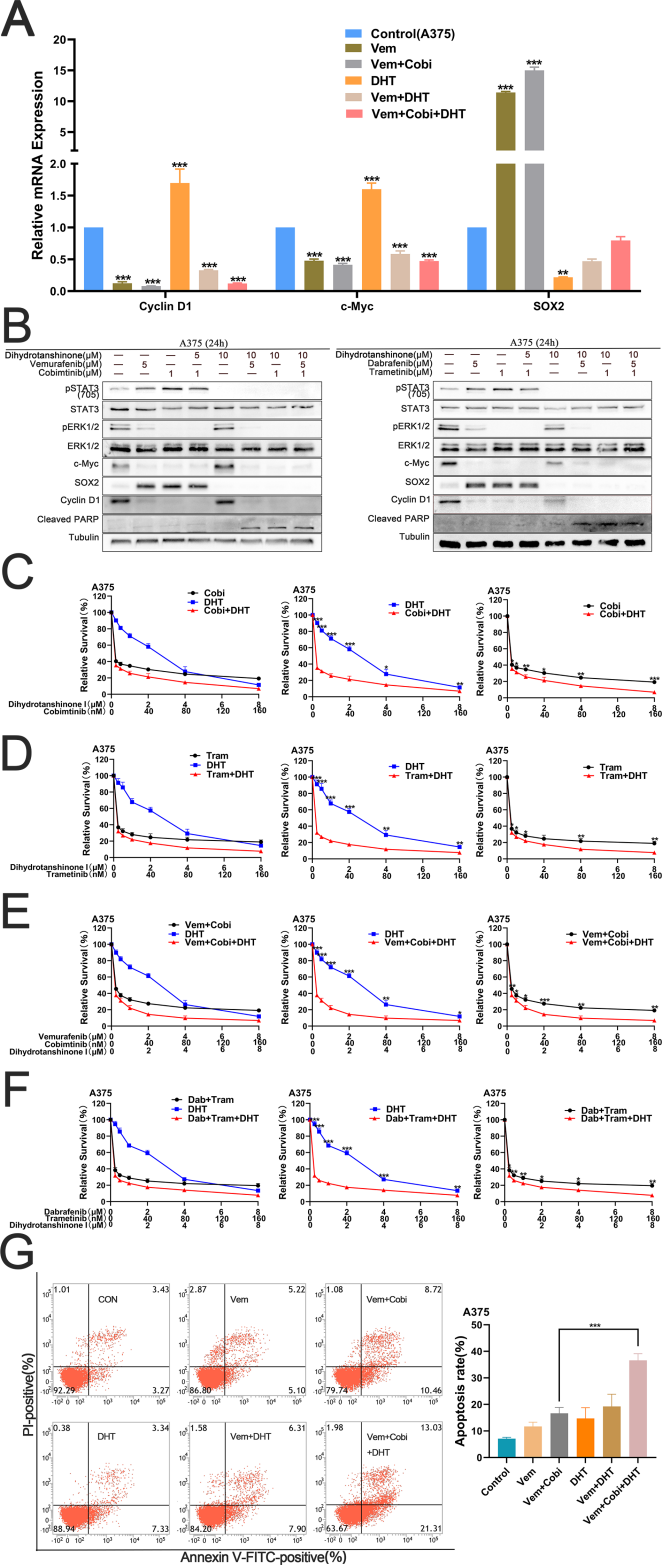
By concurrently inhibiting the MAPK and STAT3/SOX2 pathways, DHT, BRAF and MEK inhibitors can enhance the apoptosis of A375 **(A)** For 24 hours, vemurafenib (5 μM), cobimetinib (1 μM), and DHT (5 μM) were applied to A375 cells, either separately or in combination. By using real-time fluorescence quantitative PCR, the expression levels of c-Myc, SOX2, and cyclin D1 genes were identified, while β-actin served as the control. **(B)** DHT, BRAF, and MEK inhibitors (vemurafenib/cobimetinib, dabrafenib/trametinib) were administered to A375 cells for 24 hours. Using tubulin as a loading control, western blotting analysis was used to measure the expression levels of the Cleaved PARP protein, MAPK/c-Myc/cyclin D1, and STAT3/SOX2 pathways. **(C, D)** Different dosages of either cobimetinib and DHT **(C)** or trametinib and DHT **(D)** were applied to A375 cells for 72 hours. **(E, F)** Different doses of either vemurafenib, cobimetinib, and DHT **(E)** or dabrafenib, trametinib, and DHT **(F)** were added to A375 cells for 72 hours. CCK8 tests were used to measure cell viability. **(G)** A375 cells were treated with vemurafenib (5 μM), cobimetinib (1 μM), and DHT (5 μM) alone or in combination for 24 hours. Analysis of cell death in A375 cells using flow cytometry (Annexin V/PI labeling). The histogram on the right shows the proportion of dead cells. Three duplicates of each experiment were run. Data are means ± SD. ***p < 0.001; **p < 0.01; *p < 0.05; Student’s t-test.

Next, we want to find out if DHT, BRAF, and MEK inhibitors together can cause A375 cell apoptosis to occur more frequently. First, we determined the effects of various drug combinations on the RNA-level expression of the two pathways. RT-PCR results showed that STAT3 was connected to S0X2 expression, whereas c-Myc and cyclin D1 expression were connected to the MAPK pathway (**Figure 2A**). Western blot studies demonstrate that the conjunction of DHT and BRAF inhibitors, in addition to a 1μM MEK inhibitor (Cobimetinib or Trametinib) increases the inhibitory effect by blocking the MAPK and STAT3/SOX2 pathways (**Figure 2B**). Cytotoxicity tests verified that DHT, BRAF and MEK inhibitors more strongly suppressed the growth and multiplication of A375 cells (**Figures 2C-F**). Flow cytometry analysis of cell death markers similarly confirmed that the combination of three medications inhibited A375 cell activity more successfully (**Figure 2G**). According to our findings, DHT in combination with MEK and BRAF inhibitors can improve the therapeutic activity of MAPK pathway inhibitors by preventing the downstream amplification of the STAT3/SOX2 pathway. Additionally, it can also more successfully induce apoptosis in cancer cells and successfully halt BRAF mutant melanoma cells from growing and proliferating.

### 3.3 In initial drug-resistant melanoma cells, DHT with BRAF/MEK inhibitors can additionally hinder the MAPK and STAT3/SOX2 pathways at the same time, improving apoptosis

**Figure 3:**
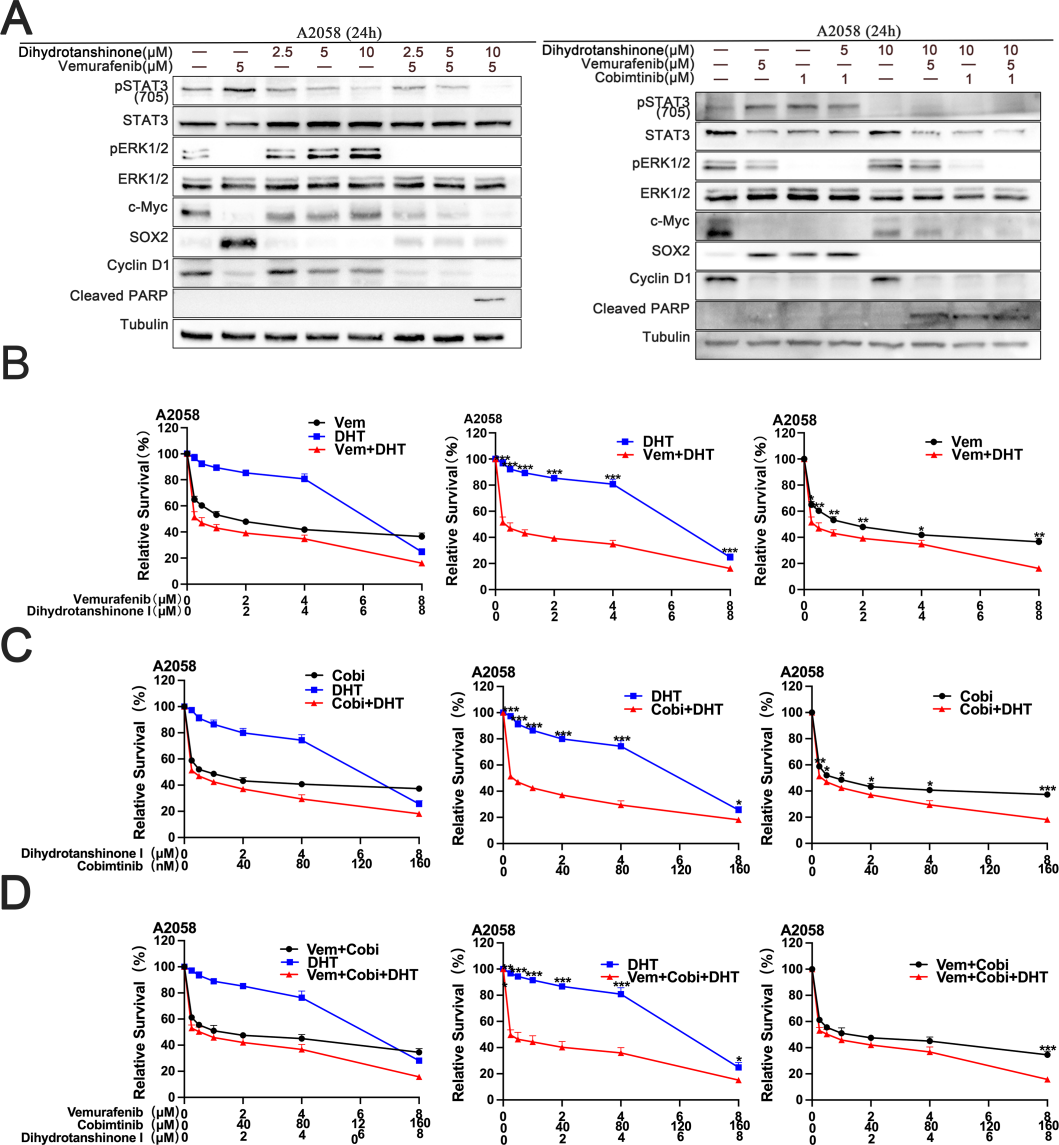
The combination of DHT and an MAPK pathway inhibitors induces apoptosis in A2058 cells **(A)** Vemurafenib and DHT or vemurafenib, cobimetinib, and DHT were administered to A2058 cells for 24 hours. Using tubulin as a loading control, western blotting analysis was used to measure the expression levels of the Cleaved PARP protein, MAPK/c-Myc/cyclin D1, and STAT3/SOX2 pathways. **(B-D)** Different concentrations of vemurafenib and DHT **(B)**, cobimetinib and DHT **(C)**, or vemurafenib, cobimetinib and DHT **(D)** were applied to A2058 cells for 72h. CCK8 tests were used to measure cell viability. There were three duplicates of each experiment. Three duplicates of each experiment were run. Data are means ± SD. ***p < 0.001; **p < 0.01; *p < 0.05; Student’s t-test.

We treated A2058 cells with DHT and BRAF/MEK inhibitors to see if DHT improves the effectiveness of MAPK inhibitors in primary drug-resistant melanoma cells. According to the experiment results, DHT and BRAF/MEK inhibitors together can inhibit the A2058 cell’s STAT3/SOX2 and MAPK pathways, which would cause the primary drug-resistant cells to undergo apoptosis (**Figure 3A**). A cytotoxicity test revealed that the three drugs together most successfully prevented A2058 cells from proliferating and growing (**Figures 3B-D**). Put differently, the conjunction of DHT and BRAF/MEK inhibitors can effectively block the pathways of STAT3/SOX2 and MAPK, decrease the growth and spread of drug-resistant primary melanoma cells, and promote apoptosis.

### 3.4 DHT and BRAF/MEK inhibitors work together to concurrently block the MAPK and STAT3/SOX2 pathways in secondary drug-resistant melanoma,thereby inducing death

**Figure 4:**
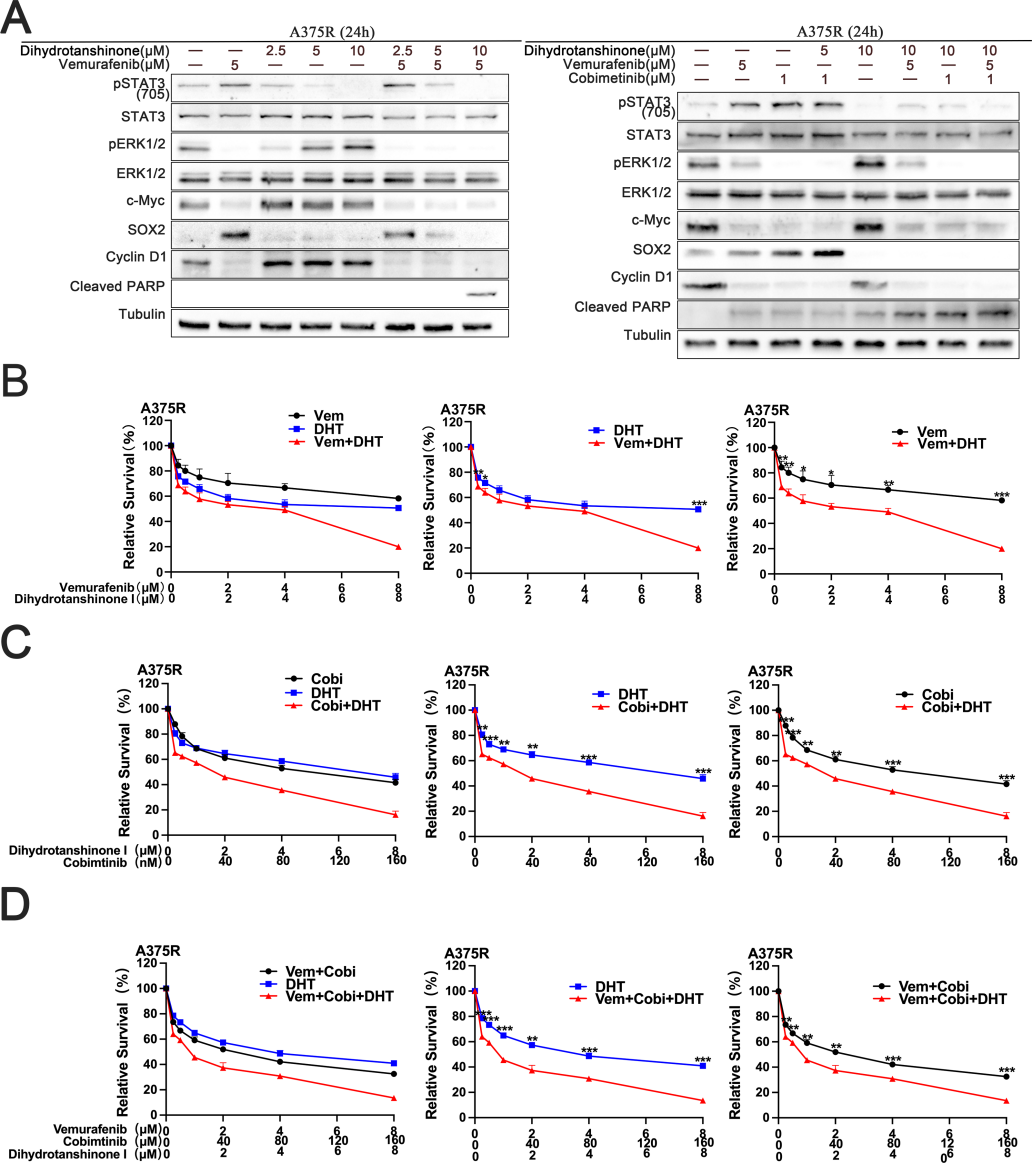
DHT augments the MAPK inhibitor’s lethal effect on A375R cells. **(A)** For 24 hours, vemurafenib and DHT or vemurafenib, cobimetinib, and DHT were administered to A375R cells. Using tubulin as a loading control, western blotting analysis was used to measure the expression levels of the Cleaved PARP protein, MAPK/c-Myc/cyclin D1, and STAT3/SOX2 pathways. **(B-D)** Different concentrations of vemurafenib and DHT **(B)**, cobimetinib and DHT **(C)**, or vemurafenib, cobimetinib and DHT **(D)** were applied to A375R cells for 72h. CCK8 tests were used to measure cell viability. Three duplicates of each experiment were run. Data are means ± SD. ***p < 0.001; **p < 0.01; *p < 0.05; Student’s t-test.

We acquired the secondary drug-resistant melanoma cell line A375R from the earlier study (7). We then investigated the inhibitory impact of DHT in combination with BRAF/MEK inhibitor on developed drug-resistant cells using A375R. The findings demonstrated that DHT in conjunction with BRAF/MEK inhibitor simultaneously inhibited the A375R cells’ STAT3/SOX2 and MAPK pathways, leading to the demise of subsequent drug-resistant cells (**Figure 4A**). A cytotoxicity test verified that the three medications together successfully slowed the growth and division of A375R cells (**Figures 4B-D**). DHT and BRAF/MEK inhibitors together may also be used to treat secondary drug-resistant melanoma cells. This combination also blocks the MAPK and STAT3/SOX2 pathways, which effectively slows or stops secondary drug-resistant melanoma cell growth and proliferation as well as causes death.

### 3.5 The conjunction of DHT and BRAF/MEK inhibitor is more likely to kill BRAF mutant melanoma cells than normal cells

**Figure 5:**
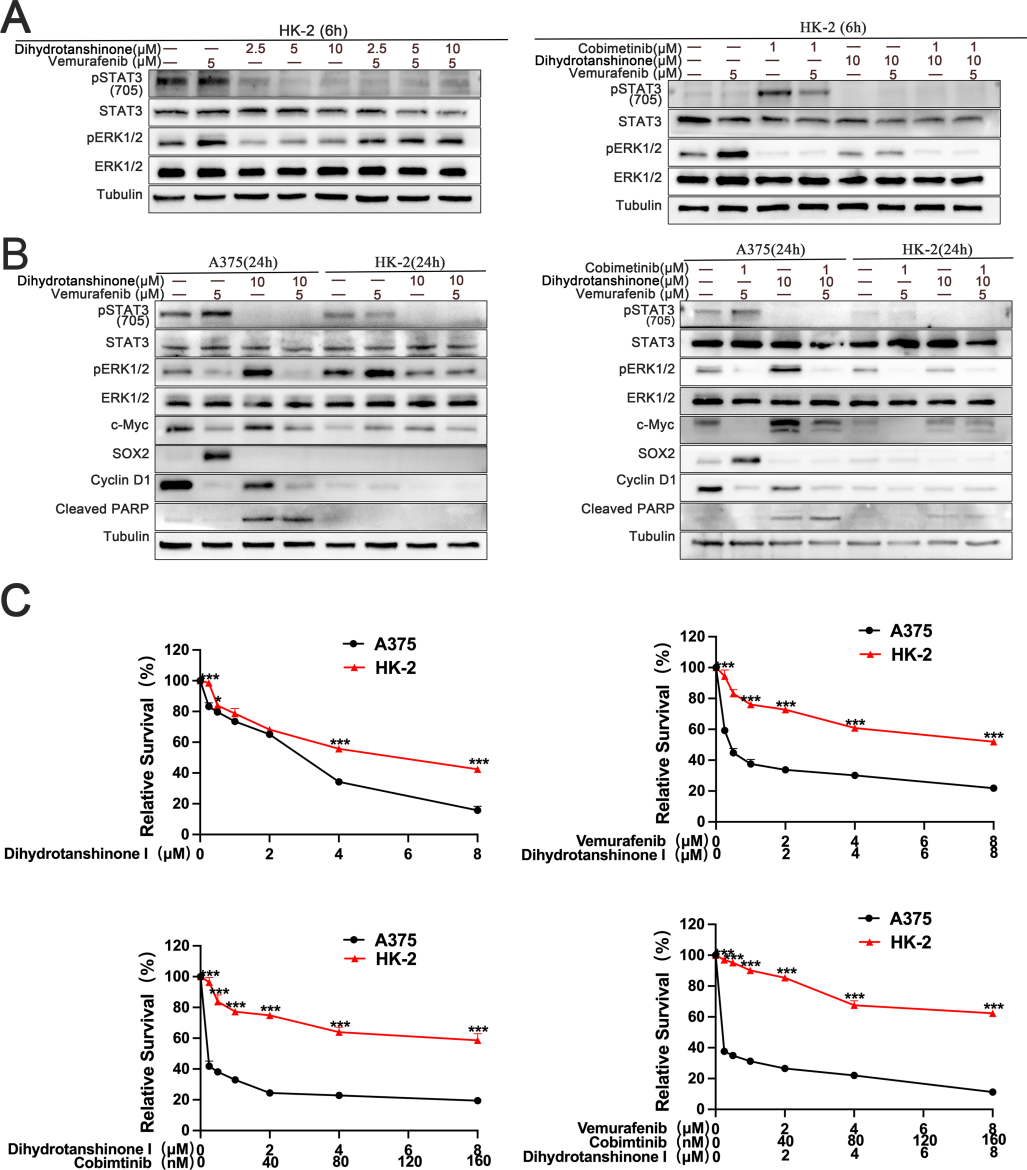
The combination of DHT and MAPK pathway inhibitor has more effective inhibitory effect on melanoma cells than Human proximaltubular epithelial cells (HK-2) **(A)** HK-2 were treated with vemurafenib and DHT or vemurafenib, cobimetinib and DHT for 6 hours. Phospho-STAT3 (705), STAT3, phospho-ERK1/2, and ERK1/2. Proteins were analyzed by western blotting, and tubulin served as a loading control. **(B)** HK-2 and A375 cells exposed to varying concentrations of vemurafenib and DHT or vemurafenib, cobimetinib and DHT for 24 hours. Using tubulin as a loading control, western blotting analysis was used to measure the expression levels of the Cleaved PARP protein, MAPK/c-Myc/cyclin D1, and STAT3/SOX2 pathways. **(C)** Different dosages of DHT and inhibitors of the MAPK pathway were applied to HK-2 and A375 cells for 72h. CCK8 tests were used to measure cell viability. Three duplicates of each experiment were run. Data are means ± SD. ***p < 0.001; **p < 0.01; *p < 0.05; Student’s t-test.

To find out how DHT and BRAF/MEK inhibitors affected normal cells, we administered different drug combinations to human proximaltubular epithelial cell line (HK-2). The findings showed that the development of two pathways in HK-2 cells could not be simultaneously inhibited by DHT and BRAF inhibitors. The three inhibitors work together to prevent the activation of two pathways in HK-2 cells at the same time (**Figures 5A, B**). The second experiment investigated the theory that BRAF mutant melanoma cells can be selectively inhibited by DHT and BRAF/MEK inhibitors combined. We looked at how DHT, either by alone or in combination with other medications, affected melanoma and normal cell apoptosis. BRAF melanoma cell death was considerably boosted by the conjunction of DHT and BRAF/MEK inhibitors as compared to HK-2 (**Figure 5C**). Thus, it can be concluded from the foregoing findings that BRAF mutant melanoma cells are more effectively inhibited by the combination of DHT and BRAF/MEK medication inhibition than normal cells are.

### 3.6 *In vivo* development and proliferation of BRAF mutant melanoma can be markedly inhibited by the conjunction of DHT and BRAF/MEK inhibitor

**Figure 6:**
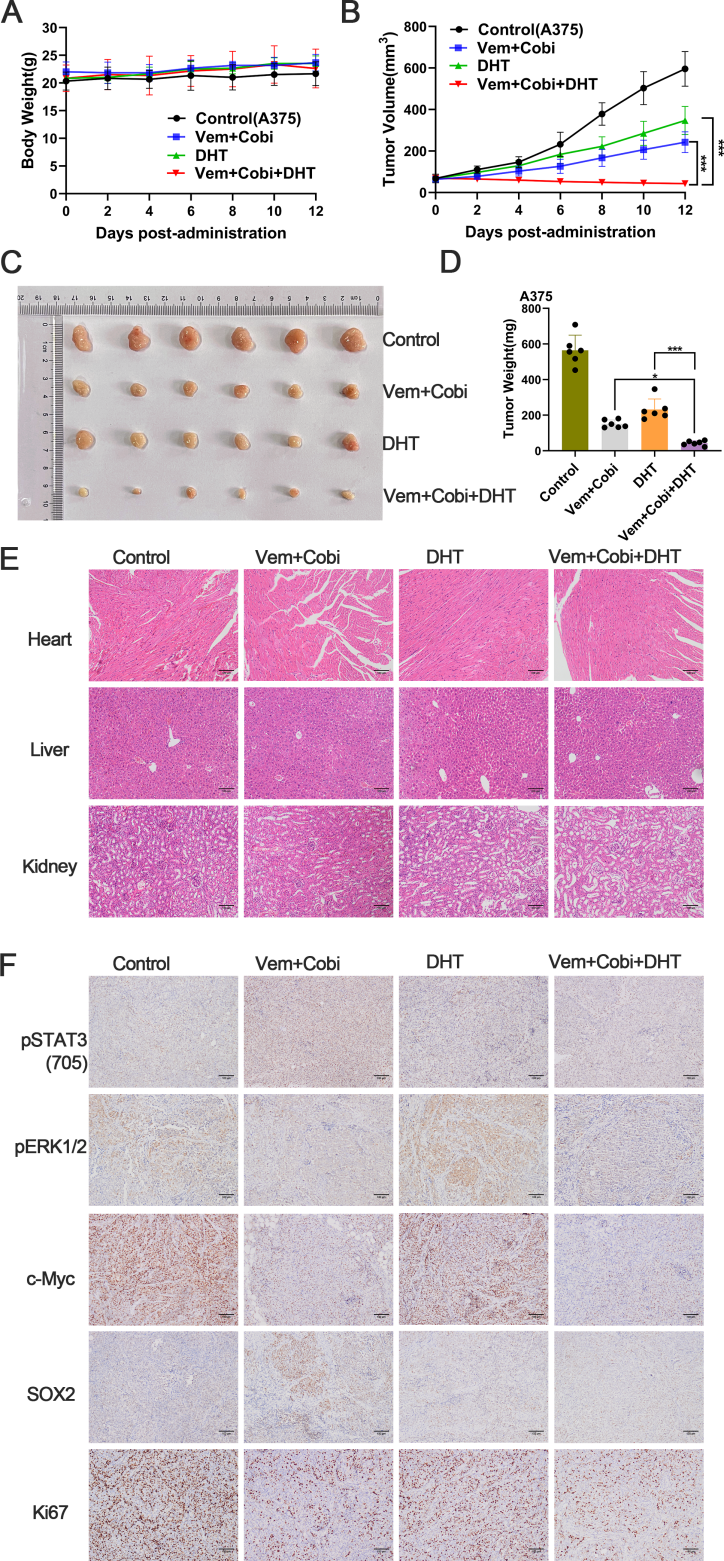
*In vivo* growth of A375 tumors can be efficiently and consistently inhibited by the conjunction of DHT and inhibitors of the MAPK pathway. **(A)** In each group of naked mice(n=6), single or combination injections of vemurafenib (Vem; 20 mg/kg), carbobimetinib (Cobi; 1 mg/kg), and DHT (DHT; 20 mg/kg) were administered. Animals were weighed every other day while receiving pharmaceutical therapy. **(B)** Every other day, the tumor size of naked mice was measured. **(C)** After 12 days of drug therapy, the A375 xenograft tumor was removed and photographed. **(D)** weighing the tumor transplant that was removed. **(E)** Haematoxylin-eosin staining of heart, liver and kidney tissue sections (magnification: ×100). **(F)** Representative images of immunohistochemical staining of p-STAT3(705)、p-ERK1/2、c-Myc、Sox2 and Ki67 in tumor tissues. The values are the mean of six separate experiments. Data are means ± SD. ***p < 0.001; **p < 0.01; *p < 0.05; Student’s t-test.

*In vitro* studies have shown that DHT suppresses the development and multiplication of BRAF mutant melanoma cells, prevents the compensatory stimulation of the STAT3 signal in these cells, and improves the therapeutic efficacy of MAPK inhibitors. However, it was unclear whether these findings were sustained *in vivo*. During the treatment period, we are assuring that the weight of mice will not be affected by the drugs, and we will give them various drug combinations (**Figure 6A**). The results demonstrated that as administration time increased, DHT and BRAF/MEK inhibitors inhibited tumor growth, with the degree of inhibition becoming increasingly apparent(**Figures 6B–D**). The results of staining the heart, liver, and kidney of mice with hematoxylin-eosin (HE) revealed that the mice were not affected by drug toxicity (**Figure 6E**). Immunohistochemical staining revealed that DHT and BRAF/MEK inhibitors inhibited STAT3/SOX2 and MAPK/c-Myc pathway expression (**Figure 6F**). According to the results of *in vivo* studies, DHT and MAPK inhibitors together showed acceptable toxicity and beneficial therapeutic efficacy when treating melanoma with a BRAF mutation.

## 4 Discussion

BRAF inhibitor-based targeted therapy, such as vemurafenib and dabrafenib, has significantly enhanced the tumor response and progression-free survival (PFS) for patients with metastatic melanoma who also have an activating BRAF mutation (19, 20). However, at least 15% of patients with advanced melanoma have a primary drug resistance mechanism, and acquired treatment resistance is nearly the same as clinical recurrence (21). Even while BRAF and MEK inhibitor combinations, including dabrafenib/trametinib and vemurafenib/cobimetinib, have shown encouraging outcomes in clinical settings, problems like acquired drug resistance and the potential for clinical side effects are still inescapable (22, 23). Consequently, the existence and emergence of drug resistance emphasises the ongoing need for combination therapy.

Combining inhibitors of the MAPK pathway with inhibitors from various pathways is a promising strategy for inhibiting drug resistance, but no effective clinical response has yet been achieved (24, 25). In BRAF-mutant melanoma cells, inhibition of the MAPK pathway may promote signal transduction and transcription activator 3 (STAT3) reactivation (7). As the convergence point of multiple carcinogenic signalling pathways, STAT3 can enhance the proliferation and migration and induce drug resistance (26, 27). Although it has been demonstrated that STAT3 is linked to the poor prognosis and treatment outcomes of various cancer types, STAT3-targeted therapy has not yet been approved for clinical use. Finding a safe and efficacious combination of STAT3 inhibitor and MAPK inhibitor to treat melanoma with BRAF mutation is therefore a potential therapeutic strategy. In the TCM Pharmacology Database and Analysis Platform (TCMSP), the drug-like property (DL) of DHT (DHT, a phenanthraquinone compound of Salvia Miltiorrhiza) was 0.36, which was greater than the reference value (0.1). The oral bioavailability of DHT was 38.75%, compared to the standard value of 20%. In the DHT group, the blood-brain barrier (BBB) was 0.39, which was greater than the reference value (0.3). Moreover, our molecular docking experiments show that DHT specifically targets the SH2 domain of STAT3 to prevent its constitutive activation. These results indicate that DHT has the potential to be an effective STAT3 inhibitor.

In melanoma, STAT3 activation causes SRY box 2 (SOX2) to be upregulated (9, 28). As a marker of cancer progenitor cells, the overexpression of SOX2 demonstrates increased BRAF inhibitor resistance (29). Our findings indicate that DHT can inhibit the release of SOX2 caused by STAT3 activation in response to BRAF inhibition. Some reports suggest that MYC may become disordered as a result of causing a mutation in the upstream RAS/RAF/MAPK pathway (30). Both C-Myc and SOX2 are reprogramming factors for inducing pluripotent stem cells (iPS), which can fuse with embryonic stem cells (ES) to reprogram them into an embryonic-like state (31). The results of our investigations verified that c-Myc and cyclin D1 expression can be decreased in BRAF mutant melanoma cells by inhibiting the MAPK pathway. Fascinatingly, this contradicts studies on hepatocellular carcinoma carried out by Jiang Xiaoli and colleagues (32).

In this study, BRAF mutant melanoma cells received treatment with a conjunction of DHT and BAAF/MEK inhibitors. The results of the experiments show that the combined therapy can block the MAPK and STAT3/SOX2 pathways’ cross-talk at the same time. We believe therefore that combination therapy can reverse the reprogramming of BRAF mutant melanoma cells, thereby overcoming drug resistance. In addition, our experimental findings demonstrate that combined therapy is equally effective on both primary and secondary drug-resistant cells, and that BRAF mutant melanoma cells are more specific than normal cells. In *in vivo* experiments, the treatment had no impact on the weight of mice, HE staining demonstrated that the drug’s toxicity was within a reasonable range, and the treatment group’s tumor size was much smaller than that of the control group. In conclusion, it has been demonstrated that the combination of DHT and BAAF/MEK inhibitor is an attractive option for treating BRAF mutant melanoma cells. However, we have not obtained clinical samples of targeted drugs for melanoma to verify our hypothesis; therefore, further discussion is required to determine whether this treatment strategy can enhance the clinical efficacy of patients and prolong their survival. In conclusion, it has been demonstrated that the conjunction of DHT and BAAF/MEK inhibitor is a promising strategy for treating BRAF mutant melanoma cells.

## Data Availability

The original contributions presented in the study are included in the article/**Supplementary Material**. Further inquiries can be directed to the corresponding authors.
