# Supplementary material for: Dihydrotanshinone I enhanced BRAF mutant melanoma treatment efficacy by inhibiting the STAT3/SOX2 signaling pathway
Source: Front Oncol. 2025 Jan 29;15:1429018. doi: 10.3389/fonc.2025.1429018 (PMC11813777; doi:10.3389/fonc.2025.1429018)
Supplement: Supplementary file 1 [file Table1.docx]

| Reagents | Company | Number |
| --- | --- | --- |
| Dihydrotanshinone I | Manstead Biotechnology | A0060 |
| Vemurafenib | Selleckchem | S1267 |
| Dabrafenib | Selleckchem | S2807 |
| Cobimetinib | Selleckchem | S8041 |
| Trametinib | Selleckchem | S2673 |

**Supplementary Table 1** The specific numbers of reagents
