# Supplementary material for: Dihydrotanshinone I enhanced BRAF mutant melanoma treatment efficacy by inhibiting the STAT3/SOX2 signaling pathway
Source: Front Oncol. 2025 Jan 29;15:1429018. doi: 10.3389/fonc.2025.1429018 (PMC11813777; doi:10.3389/fonc.2025.1429018)
Supplement: Supplementary file 2 [file Table2.docx]

| Antibodies | Company | Number |
| --- | --- | --- |
| anti-STAT3 | Abcam | ab68153 |
| anti-phospho-STAT3 | Abcam | ab76315 |
| Anti-S0X2 | Abcam | ab97959 |
| anti-CyclinD1 | CST | #2978 |
| anti-c-Myc | CST | #9402 |
| anti-cleaved PARP | CST | #5625 |
| anti-ERK1/2 | HUABIO | ER131011 |
| anti-phospho-ERK1/2 | HUABIO | ET1063-22 |
| anti-Tubulin  Anti-S0X2 | HUABIO  HUABIO | M1305-2  HA721155 |

**Supplementary Table 2** The specific numbers of reagents and antibodies
