# Supplementary material for: Dihydrotanshinone I enhanced BRAF mutant melanoma treatment efficacy by inhibiting the STAT3/SOX2 signaling pathway
Source: Front Oncol. 2025 Jan 29;15:1429018. doi: 10.3389/fonc.2025.1429018 (PMC11813777; doi:10.3389/fonc.2025.1429018)
Supplement: Supplementary file 3 [file Table3.docx]

| Gene | Forward Primer (5’-3’) | Reverse Primer (5’-3’) |
| --- | --- | --- |
| Cyclin D1 | GTCCTACTTCAAATGTGTGCAG | GGGATGGTCTCCTTCATCTTAG |
| c-Myc | CGACGAGACCTTCATCAAAAAC | CTTCTCTGAGACGAGCTTGG |
| Sox2 | AATGGGAGGGGTGCAAAAGAGG | GTGAGTGTGGATGGGATTGGTG |
| β-actin | CTCCATCCTGGCCTCGCTGT | GCTGTCACCTTCACCGTTCC |

**Supplementary Table 3** Primer sequences for RT- qPCR experiments
